# A retrospective study from a single center to compare outcomes in 79 patients with in-stent restenosis treated with paclitaxel-coated balloon angioplasty or drug-eluting stent implantation

**DOI:** 10.1186/s43044-023-00330-z

**Published:** 2023-01-31

**Authors:** Mohamed Aymen Ben Abdessalem, Anis Ghariani, Ahmed Fekih Romdhane, Fatma Ichrmad, Zied Ben Ameur, Wassim Saoudi, Hatem Bouraoui, Abdallah Mahdhaoui, Samia Ernez Hajri

**Affiliations:** Department of Cardiology, Farhat Hached University Hospital Center, Sousse, Tunisia

**Keywords:** Coronary restenosis, Drug-coated balloon, Percutaneous coronary intervention

## Abstract

**Background:**

Despite the recent progress made in drug-eluting stents (DESs), in-stent restenosis (ISR) is still a common complication of percutaneous coronary interventions. This retrospective study from a single center aimed to compare outcomes in 79 patients with ISR treated with paclitaxel-coated balloon (PCB) angioplasty or DES implantation.

**Results:**

From January 2017 to December 2021, 83 ISR lesions from 79 patients were included. Thirty-two were treated with PCB and 51 treated with available DES in the catheterization laboratory. Baseline characteristics were similar in both groups. Mean time between index angioplasty and restenosis was 27 months with a minimum of 4 months and a maximum of 70 months. Concerning Mehran ISR angiographic classification, classes II and III were more likely treated with DES. Stenosis diameter and minimal lumen diameter (MLD) were similar in both groups. PCB used was significantly shorter than DES: Mean length was 19.75 ± 5.7 versus 22.1 ± 16.5 (*p* < 0.001), respectively. Angiographic results immediately after intervention were similar in both groups: In-segment MLD after the procedure was 2.5 ± 0.4 in the DES group and 2.26 ± 0.55 in the PCB group. A median follow-up of 20 months was achieved for 68 patients, and 11 were lost to follow-up. There was also no difference in both groups regarding free from events survival.

**Conclusions:**

The findings from this study support recent international studies that have shown no significant differences between DES and PCB and in-stent restenosis. This suggests that PCB use is an option to consider in our local daily practice.

## Background

Despite the recent progress made in drug-eluting stents (DESs) [1], in-stent restenosis (ISR) is still a common complication of percutaneous coronary interventions [2]. Delayed vessel healing and neoatherosclerosis may explain that the need for repeat revascularization is still 2–3% per year [3, 4]. Treating restenosis remains challenging. Two techniques are available and validated by current guidelines: implanting a new drug-eluting stent (DES) or using a drug coated balloon in order to treat the stenosis [5]. The latter is an interesting option since it allows delivering the drug to the endothelium and, at the same time, avoids leaving a second layer of struts in the artery and, thus, avoids the chronic inflammatory response and its consequences of which are unknown [6]. However, Giacoppo et al. have suggested, in a recent meta-analysis, that DES is “moderately more effective” than paclitaxel-coated balloons (PCBs) for treating ISR [7, 8].

Even though there was sufficient data to support using PCB in ISR, its use in real life and all-comers ISR in comparison with newer generation stents as bioresorbable polymer DES and polymer-free DES still needs to be studied [9].

Therefore, this retrospective study from a single center aimed to compare outcomes in 79 patients with ISR treated with PCB angioplasty or DES implantation.

## Methods

### Ethical statement

Ethical approval was obtained from Ibn El Jazzar Medical Faculty of Sousse ethic committee (reference: CEFMS 129/2022). An informed and written consent form was signed by each patient. The consent form contained the diagnosis, the nature and aim of the recommended intervention and the expected benefits and risks of both strategies.

### Study design

This is a retrospective, observational, monocentric study of the procedural and long-term outcomes of PCB in the treatment of ISR from January 2017 to December 2021. These results were compared to ISR lesions treated with available DES during the same period. ISR was defined as a greater than 50% stenosis of a previous stented segment or up to 5 mm from the stent edges [10]. Patients with ISR who were diagnosed and treated with DES or PCB were included in the study.

### Devices

The PCB devices used included the Elutax SV paclitaxel drug-eluting balloon (Aachen Resonance, Aachen, Nordrhein–Westfalen, Germany) and the Rapid Exchange (RX) Essential paclitaxel-eluting balloon (iVascular, Barcelona, Spain) with 2.0 µg/mm^2^ paclitaxel coating designed for coronary artery use. The stents used included the Ultimaster cobalt chromium, biodegradable-polymer, sirolimus-eluting coronary stent (Terumo, Shibuya City, Tokyo, Japan), the polymer-free sirolimus-eluting coronary stent, CRE8 (Carbostent & Implantable Devices, SpA Alvimedica, Saluggia, Vercelli, Italy), and the COMBO Plus drug-eluting stent (Orbusneich, Hong Kong).

### Inclusion criteria

Patients presenting with clinical evidence of ischemic heart disease and/or a positive stress test, stable or unstable angina pectoris and a maximum of two restenosis (> 50% stenosis on visual assessment) in either bare-metal stent (BMS) or DES were eligible.

### Exclusion criteria

ST elevation myocardial infarction, patients in whom coronary artery bypass graft was indicated, lesions treated with non-drug coated balloon inflation alone, left main restenosis and evidence of thrombus in the lesion. Early restenosis happening before 3 months from the index procedure were also excluded.

### Data collection

Age, sex, cardiovascular risk factors (diabetes mellitus, hypertension, active smoking or cessation for less than 3 years, low-density lipoprotein (LDL) cholesterol level higher than 1.4 mmol/l and personal history of coronary artery disease) and left ventricular ejection fraction were all obtained from medical records.

For the initial procedural aspects, number and type of the previous stents as well as their length and diameter were collected from the patient stored records. ISR was assessed according to Mehran classification [7] by the investigators.

Angiographic lesions were assessed first by the interventional cardiologist performing the coronary angiography. Two additional cardiologists reassessed and classified the lesion according to Mehran classification [7]. Following administration of intracoronary nitroglycerine, two orthogonal projections were selected to present the target lesion free of foreshortening or vessel overlap. The lesion appeared to be the most severe at the selected projections. Quantitative coronary angiography software integrated with the Artis Zee© system Siemens Healthineers^®^ was used for quantitative analysis. Analysis were performed in-segment (area treated and 5 mm margins proximal and distal). The lesion length, reference vessel diameter, diameter stenosis, pre-procedural and post-procedure minimal lumen diameter as well as the diameter and length of the device were collected. Reference vessel diameter was defined as the computed estimation of the original diameter of the artery at the level of the obstruction. Acute lumen gain was calculated from the minimum lumen diameter (MLD) difference between post- and pre-procedure. Diameter stenosis was defined as ([1-MLD]/(Reference vessel diameter) × 100).

### Procedure

Pre-treatment with aspirin and P2Y12 inhibitors was given according to the standard practice. Anticoagulation with unfractionated heparin was administered following the local protocol. Lesion predilatation was mandated in all lesions. Choice of the diameter and length of the device was left to the operator’s discretion.

### Outcomes

A clinical follow-up was obtained in all patients in outpatient visit or by phone calls. Mean follow-up duration was 24 months. Major adverse cardiovascular event (MACE) is a composite outcome of cardiovascular death, target lesion revascularization (TLR), target vessel revascularization (TVR) and myocardial infarction (MI).

### Statistical analysis

Categorical data were presented as counts and percentages and were compared using the chi-square or Fisher’s exact test where the expected cell value was < 5. Continuous variables were analyzed for data distribution. They were either presented as mean ± standard deviation and compared using the Student’s *t* test, or as median with interquartile range and compared using the rank-sum Mann–Whitney–Wilcoxon test as appropriate. Failure rates were assessed with Kaplan–Meier analysis and compared with the log-rank test. Analyses were performed using IBM SPSS Statistics 23.

## Results

### Baseline characteristics

From January 2017 to December 2021, 83 ISR lesions from 79 patients were included. Thirty-two ISRs were treated with PCB, and 51 were treated with DES. Baseline characteristics were similar in both groups as shown in Table 1.

**Table 1:** Baseline characteristics of patients treated for in-stent restenosis with paclitaxel-coated angioplasty or drug-eluting stent

	PCB % (*n* = 32)	DES% (*n* = 51)	*p* value
Age (years)	57.9 ± 9.5	55 ± 7.3	0.79
Sex, male	65.6% (21)	82.3% (42)	0.13
Diabetes mellitus	37.5% (15)	47.7% (21)	0.18
Hypertension	75% (24)	49% (25)	0.11
Low-density lipoprotein cholesterol level higher than 1.4 mmol/l	25% (8)	35.3% (18)	0.79
Active smoker or stopped for less than 3 years	46.9% (15)	56.8% (29)	0.27
LVEF (%)	55.8% ± 6.8	56.1% ± 8.4	0.88
Personal history of coronary artery disease	25% (8)	45.1% (23)	0.19

*PCB* Paclitaxel-coated balloon, *DES* Drug-eluting stent, *LVEF* Left ventricular ejection fraction

### Clinical and angiographic characteristics of in-stent restenosis

Mean time between ISR diagnosis and the previous PCI was 27 months with a minimum of 4 months and a maximum of 70 months. No patient with 3-vessel disease was included. The characteristics of the underlying stent are resumed in Table 2. There were no chronic total occlusions nor true bifurcation lesions among the study population.

**Table 2:** Characteristics of the underlying stent used for the initial procedure of in-stent restenosis-treated patients with paclitaxel-coated balloon angioplasty or drug-eluting stent

	PCB% (*n* = 32)	DES% (*n* = 51)	*p* value
*Underlying stent*			
2 stents per lesion	40.6% (13)	9.8% (5)	**0.02**
Bare-metal stents	25% (8)	56.9% (29)	**0.033**
Drug-eluting stents	75% (24)	43.2% (22)	**0.033**
Diameter (mm)	2.93 ± 0.38	2.85 ± 0.29	0.43
Length (mm)	32.9 ± 17	25.6 ± 10.7	0.13

Bold values indicate* p* value significant (< 0.05)

*PCB* Paclitaxel-coated balloon, *DES* Drug-eluting stent

### Procedural aspects

In the DES group, only one patient had a previous coronary arteries bypass graft. ISR occurring after a BMS had been implanted in the target vessel (BMS-ISR**)** was significantly more present in the groups treated with DES, while ISR occurring after a DES had been implanted (DES-ISR) was significantly more present in the group treated with PCB. Restenosis occurring after deploying 2 stents on the same lesion was higher in the PCB group. DES used was significantly longer than PCB. ISR subsets according to Mehran’s classification, reference vessel diameter, diameter stenosis and MLD were similar in both groups. Regarding final results in both groups, MLD and acute lumen gain achieved were similar. Angiographic findings and procedural results are summarized in Table 3.

**Table 3:** Angiographic characteristics of the restenosis and procedural results of in-stent restenosis-treated patients with paclitaxel-coated balloon angioplasty or drug-eluting stent

	PCB% (*n* = 32)	DES% (*n* = 51)	*p* value
*Pre-procedure*			
Focal	34.4% (11)	27.4% (14)	0.48
Diffuse	40.6% (13)	37.2% (19)	0.57
Proliferative	9.4% (3)	29.6% (10)	0.38
Total occlusive	15.6% (5)	15.7% (8)	0.71
Lesion length (mm)	22.5 ± 7.9	24.2 ± 12.7	0.36
Reference vessel diameter (mm)	2.7 ± 0.4	2.7 ± 0.57	0.61
Used device length (mm)	19.75 ± 5.7	22.1 ± 16.5	** < 0.001**
Used device diameter (mm)	3.1 ± 0.34	2.9 ± 0.84	0.64
Minimal lumen diameter (mm)	0.93 ± 069	0.92 ± 0.59	0.80
Diameter Stenosis (%)	66.4 ± 12.4	67.9 ± 17.4	0.78
*Post-procedure*			
Minimal lumen diameter (mm)	2.26 ± 0.55	2.5 ± 0.4	0.20
Acute lumen gain (mm)	1.3 ± 0.75	1.6 ± 0.61	0.16

Bold values indicate* p* value significant (< 0.05)

*PCB* Paclitaxel-coated balloon, *DES* Drug-eluting stent

### Outcomes of treated in-stent restenosis

A median follow-up of 20 months was achieved for 68 patients, and 11 were lost to follow-up.

**Fig. 1:**
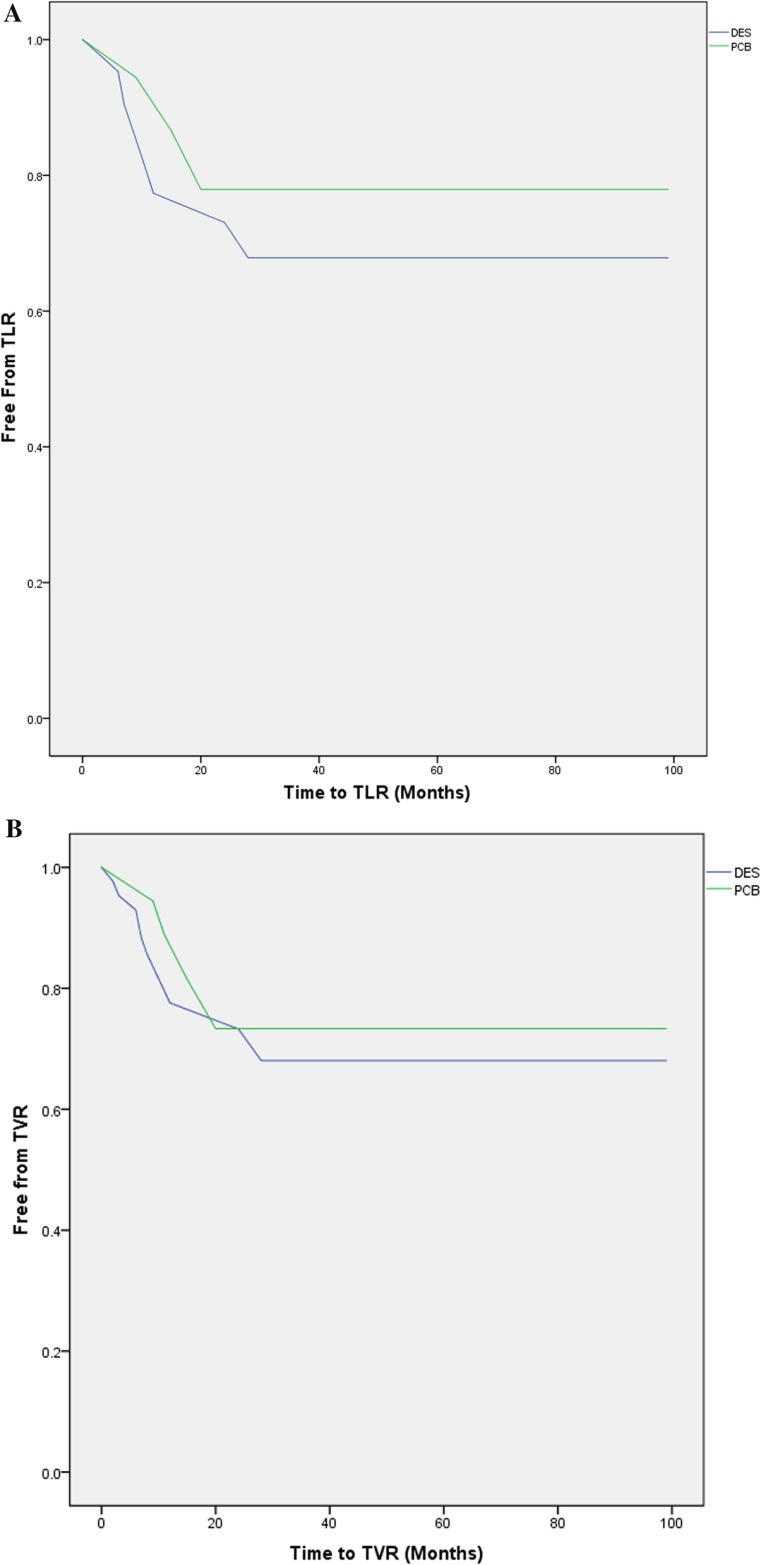

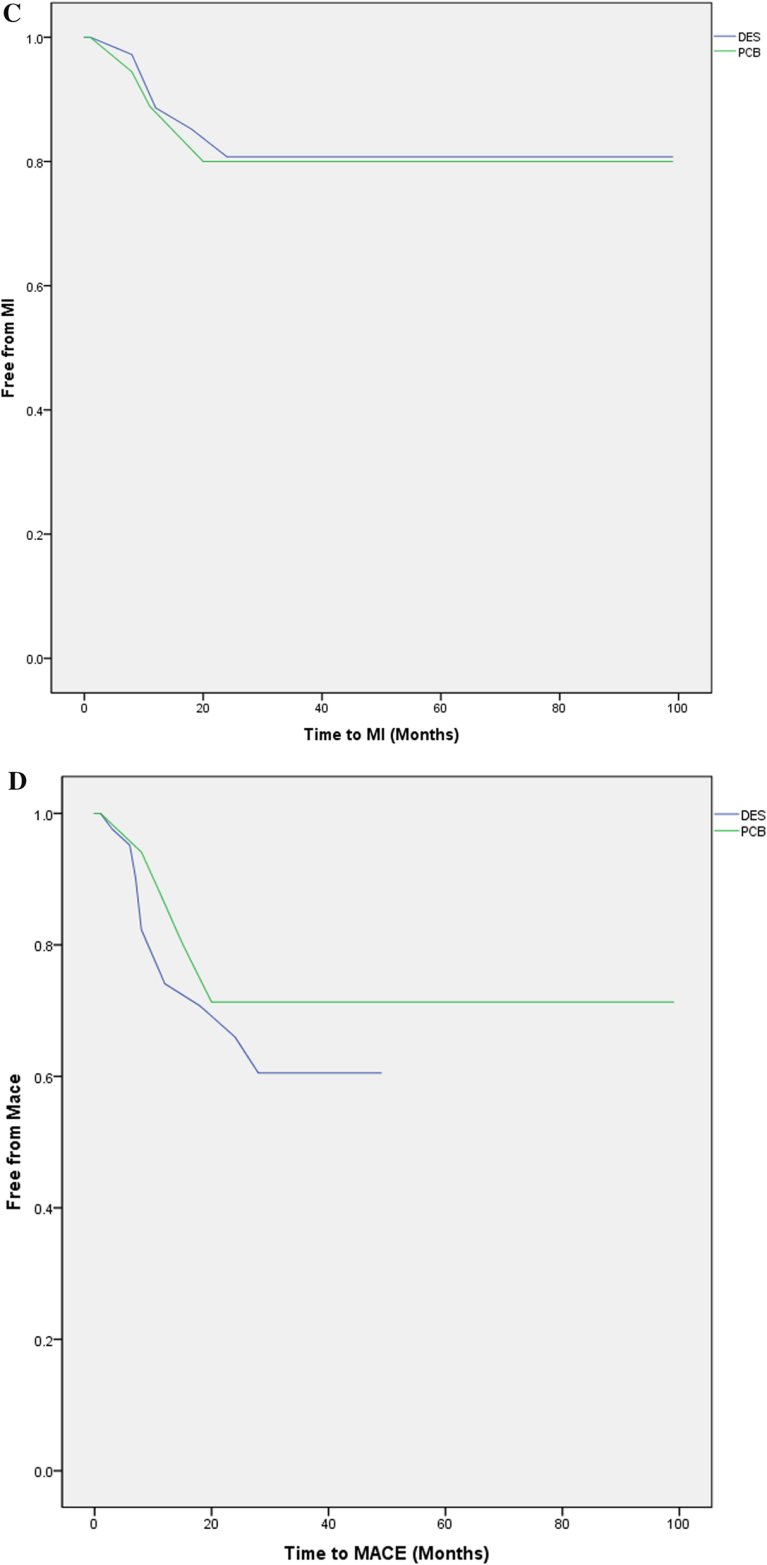
Kaplan–Meier curves demonstrating free from events rates during the follow-up period between drug-eluting stent implantation and paclitaxel-coated balloon for the treatment of in-stent restenosis. **A** Survival rates free from target lesion revascularization, **B** Survival rates free from target vessel revascularization, **C** Survival rates free from myocardial infarction, **D** Survival rates free from major adverse cardiac events. *DES* Drug-eluting stent, *MACE* Major adverse cardiac event, *MI* Myocardial infarction, *PCB* Paclitaxel-coated balloon, *TLR* Target lesion revascularization, *TVR* Target vessel revascularization; these figures have been generated with the IBM^®^ SPSS^®^ software Statistics 23

Cardiac death was not observed in both groups, TVR: 15.6% (5) versus 13.7% (7), *p* = 0.54; TLR 21.8% (7) versus 23.5% (12), p = 0.57; MI TVR: 15.6% (5) versus 13.7% (7), *p* = 0.58 and MACE: 25% (8) versus 25.5% (13), *p* = 0.9 in PCB versus DES groups, respectively. There was also no difference in both groups regarding free from events survival as provided by Kaplan–Meier curves in Fig. 1. A multivariate Cox regression analysis use of PCB was not predictor of MACE (hazard ratio 0.52; confidence interval at 95% [0.54–1.08]; *p* = 0.81).

## Discussion

Main findings of our study were that patients’ outcomes following ISR revascularization were similar whether they were treated with new generation DES or PCB as MACEs (deaths, TVR, TLR and MI) were similar in both groups.

ISR revascularization is still challenging and an evolving field of research. The current optimal approach begins with an intracoronary imaging study by optimal coherence tomography or intravascular ultrasound of the target vessel to diagnose the underlying mechanism. This imaging-guided study may distinguish between mechanical factors such as under expansion, stent fracture or recoil and biological factors such as neoatherosclerosis, and then, the appropriate technique is chosen [11]. Therapeutic arsenal contains plain old balloon angioplasty, a second DES, PCB, brachytherapy, laser therapy and plaque modification techniques such as rotablation. No intracoronary imaging was available in our context. PCB or new DES implantation was the only treatment options available. Furthermore, ISR occurring after a DES was implanted in the target vessel (DES-ISR) before 1 year of the PCI could not benefit from new DES implantation due to reimbursement issues with social services. These facts could explain why the 3/4 of the PCB were used to treat DES-ISR. While Giacoppo et al. have suggested that at 3 years, repeat stenting with DES is slightly more effective than angioplasty with PCB in reducing the need for TLR [7, 8], still wide evidence supports using both techniques to treat this type of complication [5, 12, 13]. RIBS V was one of the largest randomized controlled studies comparing PCB versus DES in BMS-ISR. Both late lumen loss and a combined outcome including TLR at a 1-year median follow-up were similar in both groups [14]. In the RIBS VI study, 309 DES-ISR patients were randomly assigned to paclitaxel-coated balloon or everolimus-eluting stent, and second groups’ results yielded less MACE 18% versus 10%, p = 0.04 principally driven from less TLR 16% versus 8% *p* = 0.035 [15]. On the other hand, in meta-analysis comparing PCB, DES and plane balloon angioplasty reported that treatment with PCB had a trend toward better outcomes than with DES [16]. DES-ISR is at high rate of recurrence and treatment is more challenging than in BMS-ISR and this could be explained by the fact that patients who already failed drug treatment are either nonresponsive or have developed drug resistance [13]. Demonstrating similar efficacy of PCB compared to DES in treating BMS-ISR is easier when we look at available data. This does not support our current practice and suggest using PCB more often in BMS-ISR than in DES. Although manufactured in more than 40 mm length within the range of diameters compatible with coronary artery disease, balloon’ lengths used in our study were significantly shorter than stents. In our practice, there is a trend to allocate PCB to focal and short diffuse restenosis. In the DARE trial [17], 278 lesions regrouping any ISR (DES or BMS in the target lesion) were allocated at 1:1 fashion to either paclitaxel-coated balloon or everolimus-eluting stent. Each type of lesion according to angiographic classification was similarly distributed in both groups *p* = 0.42. In this study, both devices were used in similar number of proliferative and occlusive restenosis 17 and 15, respectively. Furthermore, device length was also similar in both groups 22.4 ± 4.4 versus 22.1 ± 8.6, respectively, *p* = 0.72. In this study, similar results regarding maces were found [17]. In another observational retrospective cohort conducted in Italy, from the 302 lesions studied 104 were treated with PCB, and mean length of the devices used was 35.4 ± 5.2. This did not result in more procedural complication including dissection in the PCB arm. 1-year outcomes were similar in both groups. These results may suggest that using long PCB for more challenging restenosis is safe [18].

In the end, in the light of our study findings, the preferences for using PCB versus DES are resumed in Table 4 reflecting real-life practice.

**Table 4:** Preferences for using paclitaxel-coated balloon versus drug-eluting stent

Favors paclitaxel-coated balloon choice	Favors drug-eluting stent choice
Early restenosis (before 1 year)	Restenosis after 1 year
Restenosis on a drug-eluting stent	Restenosis on a bare-metal stent
Focal restenosis	Proliferative and diffuse restenosis

### Limitation

Our study was a retrospective cohort with a small number of included patients. Detailed data on the procedure preceding restenosis were not always available. Patients were not randomized which represents the most major limitation of our study.

The second major limitation was the lack of intracoronary imaging as these devices are not commonly available in our country.

## Conclusions

Despite the fact that PCB was preferably used in situations where a second DES implantation was not possible, outcomes were similar in both groups. This suggests that PCB is as efficient and safe as newer generation of DES. Our results were consistent with international studies. PCB should be an option to consider for ISR revascularization. Furthermore, it may be encouraged in BMS-ISR and in longer diffuse restenosis.

## Data Availability

The data that support the findings of this study are available from medical files stored in the Farhat Hached hospital central archive. Restrictions apply to the availability of these data, which were used under license for the current study, and so are not publicly available. Data are, however, available from the authors upon reasonable request and with permission of the chief of cardiology department.

## Abbreviations

BMS: Bare-metal stent
BMS-ISR: In-stent restenosis occurring after a bare-metal stent had been implanted
DESs: Drug-eluting stents
DES-ISR: In-stent restenosis occurring after a drug-eluting stent had been implanted
ISR: In-stent restenosis
LDL: Low-density lipoprotein
MACE: Major adverse cardiovascular event
MI: Myocardial infarction
PCB: Paclitaxel-coated balloon
TLR: Target lesion revascularization
TVR: Target vessel revascularization

## References

[1] Joner M, Finn AV, Farb A, Mont EK, Kolodgie FD, Ladich E (2006). Pathology of drug-eluting stents in humans: delayed healing and late thrombotic risk. J Am Coll Cardiol.

[2] Nakazawa G, Otsuka F, Nakano M, Vorpahl M, Yazdani SK, Ladich E (2011). The pathology of neoatherosclerosis in human coronary implants bare-metal and drug-eluting stents. J Am Coll Cardiol.

[3] Jensen LO, Thayssen P, Christiansen EH, Maeng M, Ravkilde J, Hansen KN (2016). Safety and efficacy of everolimus-versus sirolimus-eluting stents: 5-year results from SORT OUT IV. J Am Coll Cardiol.

[4] Smits PC, Vlachojannis GJ, McFadden EP, Royaards K-J, Wassing J, Joesoef KS (2015). Final 5-year follow-up of a randomized controlled Trial of everolimus- and paclitaxel-eluting stents for coronary revascularization in daily practice: the COMPARE trial (a trial of everolimus-eluting stents and paclitaxel stents for coronary revascularization in daily practice). JACC Cardiovasc Interv.

[5] Neumann F-J, Sousa-Uva M, Ahlsson A, Alfonso F, Banning AP, Benedetto U (2019). 2018 ESC/EACTS guidelines on myocardial revascularization. Eur Heart J.

[6] Buccheri D, Piraino D, Andolina G, Cortese B (2016). Understanding and managing in-stent restenosis: a review of clinical data, from pathogenesis to treatment. J Thorac Dis.

[7] Giacoppo D, Alfonso F, Xu B (2020). Drug-coated balloon angioplasty versus drug-eluting stent implantation in patients with coronary stent restenosis. J Am Coll Cardiol.

[8] Giacoppo D, Alfonso F, Xu B (2020). Paclitaxel-coated balloon angioplasty versus drug-eluting stenting for the treatment of coronary in-stent restenosis: a comprehensive, collaborative, individual patient data meta-analysis of 10 randomized clinical trials (DAEDALUS study). Eur Heart J..

[9] Jeger RV, Eccleshall S, Ahmad WAW, Ge J, Poerner TC, Shin E-S (2020). Drug-coated balloons for coronary artery disease: third report of the international DCB consensus group. JACC Cardiovasc Interv.

[10] Mehran R, Dangas G, Abizaid AS, Mintz GS, Lansky AJ, Satler LF (1999). Angiographic patterns of in-stent restenosis: classification and implications for long-term outcome. Circulation.

[11] Waksman R, Iantorno M (2018). Refractory In-stent restenosis: improving outcomes by standardizing our approach. Curr Cardiol Rep.

[12] Siontis GCM, Stefanini GG, Mavridis D, Siontis KC, Alfonso F, Pérez-Vizcayno MJ (2015). Percutaneous coronary interventional strategies for treatment of in-stent restenosis: a network meta-analysis. Lancet Lond Engl.

[13] Giacoppo D, Gargiulo G, Aruta P, Capranzano P, Tamburino C, Capodanno D (2015). Treatment strategies for coronary in-stent restenosis: systematic review and hierarchical Bayesian network meta-analysis of 24 randomised trials and 4880 patients. BMJ.

[14] Alfonso F, Pérez-Vizcayno MJ, Cárdenas A, García del Blanco B, Seidelberger B, Iñiguez A (2014). A randomized comparison of drug-eluting balloon versus everolimus-eluting stent in patients with bare-metal stent–in-Stent restenosis: the RIBS V clinical trial (Restenosis intra-stent of bare metal stents: paclitaxel-eluting Balloon vs. everolimus-eluting Stent). J Am Coll Cardiol.

[15] Alfonso F, Pérez-Vizcayno MJ, Cárdenas A, García del Blanco B, García-Touchard A, López-Minguéz JR (2015). A prospective randomized trial of drug-eluting balloons versus everolimus-eluting stents in patients with in-stent restenosis of drug-eluting stents: the RIBS IV randomized clinical trial. J Am Coll Cardiol.

[16] Lee JM, Park J, Kang J, Jeon K-H, Jung J, Lee SE (2015). Comparison among drug-eluting balloon, drug-eluting stent, and plain balloon angioplasty for the treatment of in-stent restenosis: a network meta-analysis of 11 randomized Controlled Trials. JACC Cardiovasc Interv.

[17] Baan J, Claessen BE, Dijk KB, Vendrik J, van der Schaaf RJ, Meuwissen M (2018). A randomized comparison of paclitaxel-eluting balloon versus everolimus-eluting stent for the treatment of any in-stent restenosis: the DARE trial. JACC Cardiovasc Interv.

[18] Basavarajaiah S, Naganuma T, Latib A, Sticchi A, Ciconte G, Panoulas V (2016). Treatment of drug-eluting stent restenosis: comparison between drug-eluting balloon versus second-generation drug-eluting stents from a retrospective observational study. Catheter Cardiovasc Interv.

